# Fucoidan Exerts Anticancer Effects Against Head and Neck Squamous Cell Carcinoma In Vitro

**DOI:** 10.3390/molecules23123302

**Published:** 2018-12-12

**Authors:** Wiktoria Blaszczak, Michal Stefan Lach, Wojciech Barczak, Wiktoria Maria Suchorska

**Affiliations:** 1 Radiobiology Lab, The Greater Poland Cancer Centre, Garbary 15 Str., 61-866 Poznan, Poland; w.z.blaszczak@gmail.com (W.B.); michal.lach@wco.pl (M.S.L.); wiktoria.suchorska@wco.pl (W.M.S.); 2 Department of Electroradiology, Poznan University of Medical Sciences, Garbary 15 Str., 61-866 Poznan, Poland; 3 Postgraduate School of Molecular Medicine, Warsaw University of Medical Sciences, Ks. Trojdena 2a Str., 02-109 Warsaw, Poland; 4 Department of Head and Neck Surgery, Poznan University of Medical Sciences, The Greater Poland Cancer Centre, Garbary 15 Str., 61-866 Poznan, Poland

**Keywords:** fucoidan, head and neck cancer, marine algae, complementary therapy, HNSCC

## Abstract

Fucoidans have been reported to exert anticancer effects with simultaneous low toxicity against healthy tissue. That correlation was observed in several cancer models, however, it has never been investigated in head and neck cancer before. To magnify the efficacy of conventional therapy, the administration of agents like fucoidan could be beneficial. The aim of this study was to evaluate the anticancer effect of *Fucus vesiculosus* (FV) extract alone and with co-administration of cisplatin in head and neck squamous cell carcinoma (HNSCC) in vitro. MTT assay results revealed an FV-induced inhibition of proliferation in all tested cell lines (H103, FaDu, KB). Flow cytometric cell cycle analysis showed an FV-induced, dose-dependent arrest in either S/G2 phase (H103, FaDu) or G1 arrest (KB). Furthermore, a dose-dependent gain in apoptotic fraction was observed. Western blot analysis confirmed the induction of apoptosis. A significant dose-dependent increase in reactive oxygen species (ROS) production was revealed in the H103 cell line, while FaDu cells remained unresponsive. On the contrary, an HPV-positive cell line, KB, demonstrated a dose-dependent decrease in ROS synthesis. Moreover, fucoidan enhanced the response to cisplatin (synergistic effect) in all cell lines with the HPV-positive one (KB) being the most sensitive. These results have been confirmed by flow-cytometric apoptosis analysis. In conclusion, we confirmed that fucoidan exhibits anticancer properties against HNSCC, which are manifested by the induction of apoptosis, regulation of ROS production, cell cycle arrest, and inhibition of proliferation.

## 1. Introduction

Fucoidan is a sulfated fucose-rich polysaccharide derived from cell membranes of marine algae species such as *Fucus* spp. and *Undaria* spp. [1]. Its therapeutic properties have been extensively studied throughout the last decades with a strong emphasis on anticancer activity [2]. Various in vitro studies revealed its capacity to induce apoptosis [3,4] and inhibit angiogenesis [5] and the proliferation of cancer cells [6,7]. The anticancer potential of fucoidan has also been reported in vivo, where it significantly decreased tumor size and inhibited metastasis [8,9]. Moreover, fucoidan is well tolerated, even at high doses, and does not trigger the incidence of adverse effects [10]. However, the beneficial properties of fucoidans are variable throughout the whole group of fucans. They are dependent mostly on the species from which the fucoidan has been derived, the extraction method, and the structure and chemical composition of polysaccharide, especially its degree of sulfation. There are also reports suggesting that the effects of fucoidan might be cell-dependent [11].

Head and neck squamous cell carcinoma (HNSCC), as the sixth most common cancer worldwide, still poses a challenge for clinicians with its 5-year survival rate not exceeding 50% [12]. HNSCC has been reported to be associated with excessive use of alcohol, tobacco, Human Papilloma Virus (HPV), and Epstein-Barr Virus (EBV) infection [13,14]. The standard treatment of HNSCC includes surgical resection of the tumor and radio- and/or chemotherapy, a treatment which tends to not be well tolerated and often leads to severe adverse effects, which might undermine the continuation of the therapy according to the recommended protocols [15,16]. Therefore, the interest in agents, such as fucoidan, that could enhance the beneficial effects of conventional treatment or reduce the frequency of serious adverse effects has arisen.

The aim of this study was to assess the effects of crude *Fucus vesiculosus*-derived fucoidan on head and neck squamous cell carcinoma in vitro, including the impact on cell cycle progression, proliferation rate, apoptosis, reactive oxygen species (ROS) production, and possible alteration of effects yielded by cisplatin administration.

## 2. Results

### 2.1. Fucoidan Inhibits Proliferation of HNSCC In Vitro

**Figure 1:**
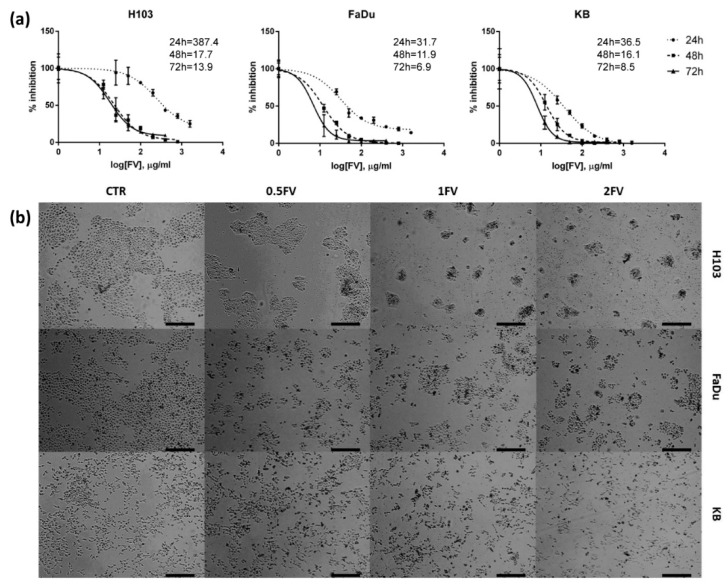
Effect of fucoidan on the proliferation and viability of HNSCC cell lines: H103, FaDu, KB. (**a**) Inhibition of proliferation of tested cell lines after incubation with fucoidan for 24, 48 and 72 h. The data are presented as a mean ± standard deviation. (**b**) The decreased proliferation of tested cell lines after 24 h incubation with different concentrations of fucoidan was observed (CTR—control, 0.5FV—half-fold of IC50 concentration, 1FV—IC50 concentration, 2FV—2-fold of IC50 concentration). Pictures were taken under 40× magnification. Scale bars represent 200 µm.

Incubation with increasing doses of fucoidan representing 0.5-; 1- and 2-fold of IC50 (0.5FV, 1FV, 2FV) for 24 h revealed that FV inhibits the proliferation of H103, FaDu, and KB cell lines in a dose-dependent manner irrespective of HPV infection status. Extending the incubation period to 48 h and 72 h magnifies the observed inhibition by even lower concentrations (Figure 1a,b).

### 2.2. Treatment With Fucoidan Leads to Cell Cycle Perturbations Causing Arrest in G2/S or G1 Phase in HNSCC Cells

**Figure 2:**
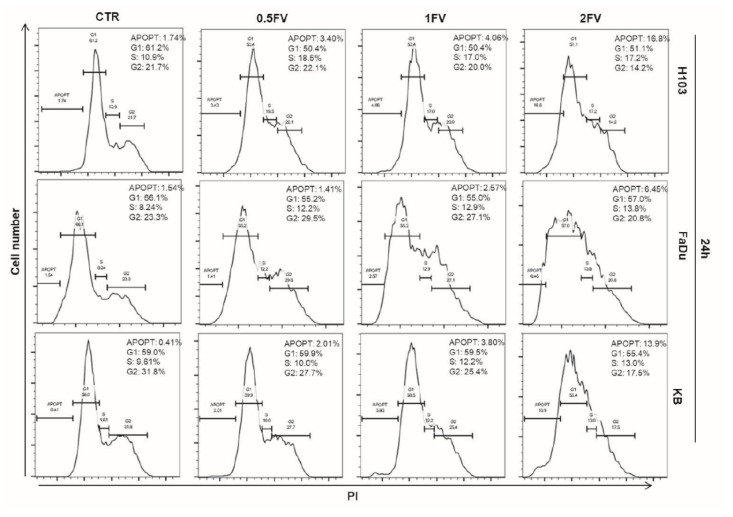
Impact of fucoidan on cell cycle distribution of HNSCC cell lines: H103, FaDu, KB. Cell cycle analysis after incubation with different concentrations of fucoidan for 24 h. Increasing dose of fucoidan increases the number of apoptotic cells in all tested cell lines. Treatment with fucoidan induces G2/S arrest in H103 and FaDu cells, and G1 arrest in KB cell line. (CTR—control, 0.5FV—half-fold of IC50 concentration, 1FV—IC50 concentration, 2FV—2-fold of IC50 concentration, PI—propidium iodide).

Fucoidan impacts cell cycle distribution in all tested cell lines and induces cell cycle arrest in the G2/S phase in H103 and FaDu cells in a dose-dependent manner. In the case of KB cell line, we observed a slight FV-induced G1 phase arrest. What is more, incubation with fucoidan increased the apoptotic fraction in a dose-dependent manner in all HNSCC cell lines, which was represented by the increased percentage of the population of cells in the sub-G1 phase (Figure 2).

### 2.3. Fucoidan Induces Apoptosis of HNSCC Cells Due to the Regulation of ROS Production

**Figure 3:**
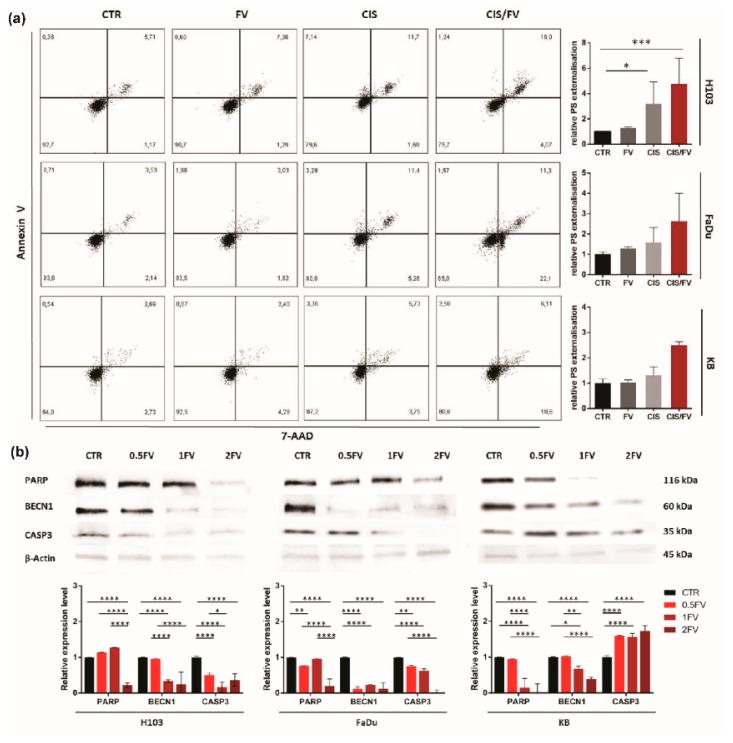
Impact of fucoidan on the induction of apoptosis in HNSCC cell lines: H103, FaDu, KB (**a**): 24 h incubation with fucoidan induces apoptosis in all tested cell lines (CTR—control, 0.5FV—half-fold of IC50 concentration, 1FV—IC50 concentration, 2FV—2-fold of IC50 concentration, PS—phosphatidylserine). The data are presented as an average ± standard deviation. (**b**): 24 h treatment with fucoidan down-regulates the levels of full-length PARP, BECN1, and CASP3 in H103 and FaDu cells. Fucoidan decreases the expression of PARP and BECN1 while up-regulating levels of CASP3 in KB cell line. (CTR—control, 0.5FV—half-fold of IC50 concentration, 1FV—IC50 concentration, 2FV—2-fold of IC50 concentration). The data are presented as mean ± standard deviation. *p* values lower than 0.05 were considered significant and are labeled by asterisks (*) for *p* < 0.05, (**) for *p* < 0.01, (***) for *p* < 0.001, and (****) for *p* ≤ 0.0001.

**Figure 4:**
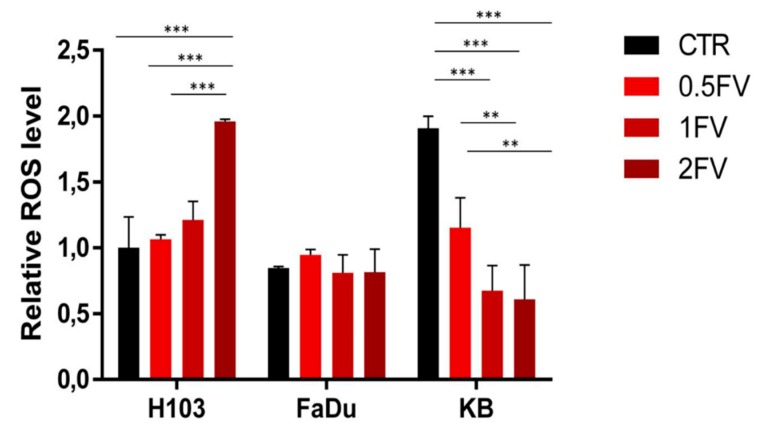
Impact of fucoidan on the synthesis of reactive oxygen species in HNSCC cells. Twenty-four hour treatment with fucoidan up-regulates ROS production in a dose-dependent manner in H103 cells, whilst down-regulating it in KB cells. No effect was observed on FaDu cells. (CTR—control, 0.5FV—half-fold of IC50 concentration, 1FV—IC50 concentration, 2FV—2-fold of IC50 concentration). The data are presented as an average ± standard deviation.

Flow cytometric analysis revealed FV-induced externalization of phosphatidylserine in all HNSCC cell lines. The effect was dose-dependent, and the most sensitive cell line was H103 (Figure 3a). Additionally, induction of apoptosis was confirmed by Western blot, which revealed a down-regulation in the full-length forms of PARP and CASP3 in H103 and FaDu following 24 h treatment with FV. The effects observed in KB cells differ regarding CASP3, in this case, fucoidan treatment correlated with up-regulated expression (Figure 3b). Additionally, in all tested cell lines, the down-regulation of BECN1 (an anti-apoptotic marker) was observed, which indicates direct caspase-dependent apoptosis is occurring. Furthermore, 24 h treatment with fucoidan increased ROS production in H103 cells. In contrast, an opposite effect was observed in KB cells, where incubation with fucoidan reduced ROS production. Both effects were dose-dependent. However, FaDu cells remained unresponsive to fucoidan, as ROS production was not altered after treatment, which suggests that cell death was not related to oxidative stress but rather to direct activation of caspase-dependent apoptosis (Figure 4).

### 2.4. FV Enhances Response to Cisplatin

**Figure 5:**
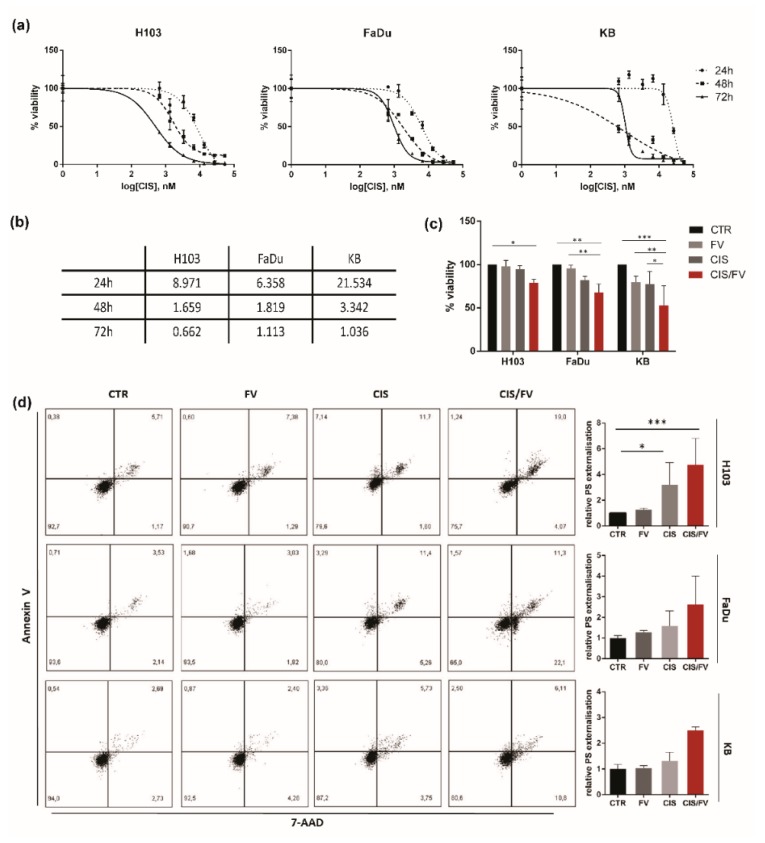
Simultaneous co-administration of cisplatin and fucoidan on HNSCC cell lines: H103, FaDu, KB. (**a**) Survival curves of HNSCC cells generated after exposure to cisplatin for 24, 48 and 72 h. (**b**): IC50 doses after 24, 48 and 72 h incubation with cisplatin in µM. (**c**) Fucoidan enhances the response to cisplatin by further decreasing the proliferation of all tested cell lines after 8 h incubation (H103) and 16 h (FaDu, KB) at the IC50 concentrations. The data are presented as mean ± standard deviation. (**d**) Fucoidan enhances the response to cisplatin by further inducing apoptosis of all tested cell lines after 8 h incubation (H103) and 16 h (FaDu, KB) at the IC50 concentrations. CTR—control, CIS—cisplatin, CIS/FV—cisplatin and fucoidan, PS—phosphatidylserine). *p* values lower than 0.05 were considered significant and are labelled by asterisks (*) for *p* < 0.05, (**) for *p* < 0.01, (***) for *p* < 0.001, and (****) for *p* ≤ 0.0001.

In order to assess a possible alteration of response to cisplatin induced by simultaneous co-administration of fucoidan, an MTT assay using cisplatin at concentration ranges between 0.667 µM to 53.36 µM was performed, and IC50 doses were calculated (Figure 5a,b). To establish an optimal time point for further analysis, cells were treated with the respective IC50 concentration of cisplatin for 2, 4, 6, 8, 16 and 24 h. Obtained MTT results proved that an 8 h incubation with cisplatin in the case of H103 cells, and 16 h in both FaDu and KB cells, yielded no toxicity. Thus, cells were treated with the respective dose of cisplatin alone or in combination with fucoidan (IC50 doses) for 8 h (H103) or 16 h (FaDu, KB). We found that simultaneous administration of cisplatin and fucoidan magnifies toxicity and lowers cell viability in all HNSCC cell lines (Figure 5c). These data were confirmed by flow cytometric analysis of apoptosis, although significant changes were observed only in H103 cells (Figure 5d). To assess in detail the character of fucoidan and cisplatin activity, we conducted an analysis of drug interactions. This analysis revealed a synergy between FV and CIS in all tested cell lines in both experiments, with the exception of FaDu cells. In this case, analysis of apoptosis in cells receiving combined treatment demonstrated an additive effect (Table 1).

## 3. Discussion

The anticancer activity of therapeutics relies mostly on their antiproliferative and proapoptotic properties, which enable successful eradication of cancer cells [17]. In this study, we proved that treatment with crude *Fucus vesiculosus*-derived fucoidan inhibits the proliferation of HNSCC cell lines in a dose- and time-dependent manner. Our results are in line with data obtained by Ale, Xue, and Hyun, who observed antiproliferative effects of fucoidan on breast cancer, colorectal cancer, and melanoma respectively [10,18,19].

One of the hallmarks of cancer cells is their high proliferative rate. The mechanism behind this phenomenon is complex and is associated with mutations acquired during tumorigenesis, especially those affecting cell cycle regulatory genes, such as RB and TP53, which allows for a cell to escape the control mechanisms, and by that, stimulate its unlimited divisions [20]. As a result, a rapid increase of tumor mass is observed, which corresponds with poor prognosis. Therefore, therapeutic approaches often target pathways deregulated in cancer to induce cell cycle arrest and decrease the proliferation rate of cancer cells [21]. In this study, we found that fucoidan induced cell cycle arrest in the G1 (KB) or G2/S phase (H103, FaDu). Similar to our observations, it has previously been proven that the treatment of cells with fucoidan of different origin induced cell cycle arrest in various phases [22,23]. Banafa et al., performed studies on breast cancer cells, where they observed a *Fucus vesiculosus*-induced arrest in G1 phase following a 24 h incubation [23]. Similar results were obtained by Han et al., who tested *Fucus vesiculosus* extract’s capacity to deregulate the cell cycle of HT29 colorectal cancer cells. Researchers observed a greater than 20% increase in G1 phase cells after incubation with fucoidan [24]. We did not observe such vivid changes in G1 fraction in our study. We conclude that FV leads to G1 arrest in KB cells based on the stability of the G1 fraction upon increasing the dose of fucoidan. Moreover, we observed a simultaneous decrease in G2 and an increase in the sub-G1 fraction. The differences in efficacy of fucoidan to induce cell cycle arrest might stem from the large differences in the dose used between our study and theirs. Banafa’s research utilized doses of fucoidan up to 1000 µg/mL, while in our study, KB cells were treated with lower concentrations: 18.25 µg/mL (0.5FV), 36.50 µg/mL (1FV) and 73.00 µg/mL (2FV). We may also speculate that the effects induced by fucoidan are cell type and HPV-status dependent. There is no data in the literature on FV-induced G2/S phase arrest. This might be due to the fact that the effects of fucoidan on head and neck cancer have not been tested previously, and such arrest results from specific HNSCC characteristics. This belief is supported by data obtained by Hu et al., who observed G2/S phase arrest in HNSCC cell lines (SCC-15 and FaDu) following treatment with curcumin [25].

The increased numbers of cells observed in the sub-G1 fraction during cell cycle assessment following incubation with fucoidan have been confirmed by the analysis of induction of apoptosis. We proved that fucoidan-based treatment effectively induced apoptosis in HNSCC cell lines, irrespective of HPV infection status. Our findings are in line with results obtained in different studies. Jinglong et al., in their research on the response of RPMI8226 and U266 cells (multiple myeloma model) to fucoidan, observed an increase in the number of apoptotic cells after 72 h incubation with 12.5 µg/mL fucoidan [26]. Similar results were found by Aisa et al., who reported a gain in apoptotic fraction after 48 h incubation of HS-Sultan cells with 100 µg/mL of *Fucus vesiculosus* extract [27]. Pro-apoptotic activity of fucoidan has also been observed in a study conducted on a colorectal cancer model. Kim et al., reported that concentrations of fucoidan as low as 20 µg/mL induce apoptosis of HT-29 and HCT116 cells [28]. Analogous results were demonstrated in research conducted on lung [9], bladder [29] and ovary cancer models [30], however, fucoidan was ineffective in studies carried out on uveal melanoma [31]. Our Western blot analysis revealed a down-regulation of full-length PARP and full-length CASP3 in both H103 and FaDu cell lines, after 24 h treatment with FV. A similar dose-dependent loss of full-length PARP following treatment with fucoidan has been reported by Park et al., and Hsu et al., [29,32]. Also, a dose-dependent down-regulation in the expression of the full-length form of CASP3 has been observed by Lee et al., which proves that apoptosis induced by fucoidan treatment is caspase-dependent [33]. The effects observed in KB cells differ regarding CASP3, in this case, fucoidan treatment correlated with up-regulated expression, which might stem from the cell line’s HPV infection status.

Reactive oxygen species as regulatory molecules are crucial in cell signaling. ROS are elements of various signaling pathways, and impact on the regulation of apoptosis (especially the extrinsic pathway), proliferation, differentiation, as well as enable the respiratory burst in phagocytes [34]. However, it was proven that in relation to reactive oxygen species the activity of fucoidan might be diverse [35]. In this study, we found that 24 h incubation with fucoidan up-regulates intracellular ROS production in a dose-dependent manner in H103 cells. An opposite effect was observed in the KB cell line, whilst in FaDu cells, ROS levels remained unaltered. Our findings seem to confirm diverse activity of fucoidan in regard to ROS synthesis. Han et al., found that treatment with fucoidan up-regulated ROS production in bladder cancer cells (5637), which corresponded with the induction of apoptosis. Moreover, the effect was neutralized by the addition of an antioxidant to fucoidan-treated cells [4]. Similar results have been obtained by Banafa et al., who observed an increase in ROS levels in a breast cancer model following treatment with *Fucus vesiculosus*-derived fucoidan [23]. Fucoidan of the same origin was also reported to efficiently stimulate ROS production in hepatocellular carcinoma cells [36]. Moreover, administration of fucoidan elevated ROS levels in Non-Small-Cell Lung Carcinoma (NSCLC), where ROS production increased 10 min after treatment, reaching its peak after 30 min [37]. However, incubation with fucoidan under hypoxic conditions decreased reactive oxygen species production in bladder cancer cells, therefore restoring their physiological level [38].

The extent of ROS synthesis might be deregulated due to ongoing HPV infection. It was proven that E2 oncoprotein activation subsequent to HPV type 18 infection stimulates reactive oxygen species production and stabilizes it at a higher level than observed in healthy cells. Mostly, this is caused by increased release of ROS from mitochondria and the simultaneous down-regulated expression of SOD1 and glutathione [39]. Moreover, concurrent expression of E6 and E7 elevates ROS levels in HNSCC cells [40]. Therefore, we conclude that the observed up-regulated ROS production in untreated KB cells and its reduction after fucoidan administration correlates with literature data and suggests that fucoidan attenuates HPV-induced deregulation in ROS synthesis. However, the activity and efficacy of fucoidan depends on the type of cells and culture conditions (mostly oxygen concentration). As ROS production increased in H103 cells treated with fucoidan, and this correlated with decreased expression of full-length CASP3 and PARP (neither of which was reported in KB cells), we conclude that caspase activation (in H103 cells) has been triggered by oxidative stress.

One of the most common approaches in HNSCC therapy is surgical resection of the tumor, followed by radio- and/or chemotherapy. For the majority of cases, the standard procedure includes platin-based chemotherapy and subsequent irradiation [41]. Unfortunately, the activity of cisplatin is not specific towards cancer cells, which are more susceptible than healthy counterparts only because of their higher proliferation rate [42]. This lack of selective targeting corresponds with high toxicity of therapy and the incidence of serious adverse effects, which not only decreases the quality of life but might trigger severe complications [43]. Hence, the interest in agents that might either magnify the beneficial effects of conventional therapy or reduce the incidence of adverse effects has arisen. Thus far, several agents effective in enhancing the response to cisplatin have emerged, and include apigenin [44], resveratrol [45], and extracts of *Nauclea orientalis*, *C. vincetoxicum* and *T. tanakae* [46]. In our study, altered response to cisplatin after simultaneous administration with fucoidan has been investigated. We observed that co-administration of fucoidan to cisplatin-treated cells magnified the therapeutic effect in all tested cell lines, however, the combination was most effective in KB cells. Depending on the type of conducted analysis, we detected the enhancement of response to differing extents, which was a result of the different nature of both tests. Furthermore, subsequent analysis of the drug combinatory effect revealed a synergy between fucoidan and cisplatin in all tested HNSCC cell lines. Similar results have been obtained by Zhang et al., who researched the effects yielded by common cytostatics: cisplatin, paclitaxel, tamoxifen, and a combination of these agents with fucoidan. The authors reported that combined use of cytostatics and fucoidan yielded greater therapeutic effects in comparison to single agent treatment. They also reported that the combination of fucoidan and cisplatin was the most effective one [47]. The results have been confirmed by Abuddabus et al., who found that simultaneous incubation of cisplatin, doxorubicin or paclitaxel with fucoidan magnified therapeutic effects when compared to single agents [48]. A synergy between fucoidan and lapatinib has also been reported [49].

## 4. Materials and Methods

### 4.1. Fucoidan and Cisplatin

Crude fucoidan derived from *Fucus vesiculosus* (FV) was obtained from Sigma Aldrich (F5631, Sigma Aldrich, St. Louis, MO, USA). Five-hundred miligrams of dry FV was dissolved in 5 mL PBS and stored in −20 °C. An aqueous solution of cisplatin (Teva Pharmaceutical Industries Ltd., Petach Tikwa, Israel) at the concentration of 1 mg/mL was stored at room temperature in the dark for future analysis.

### 4.2. Cell Culture

The study was conducted using three HNSCC cell lines: H103 (ECACC, no. 06092001), FaDu (ATCC^®^, HTB-43™), and HPV positive—KB (ATCC^®^, CCL-17™). FaDu and KB cell lines were cultured in DMEM (Biowest, Nuaillé, France) supplemented with 10% fetal bovine serum (Biowest, France) and 1% of penicillin and streptomycin mixture (Merck, Darmstadt, Germany); H103 cells were cultured in 1:1 DMEM and Ham’s F12 (Biowest, France) nutrient medium with 10% fetal bovine serum, 1% of L-glutamine (Biowest, France) and 1% of penicillin and streptomycin mixture. All cells were cultured at 37 °C with 100% humidity at the 5% CO2 atmosphere.

### 4.3. MTT Assay

To assess the cytotoxic effect of FV on HNSCC cells, the culture was seeded into 96-well culture plates at the desired density (Table 2). Next, cells were incubated with FV of variable serial concentrations (24 h: 25–1600 μg/mL, 48 h: 12.5–800 μg/mL, 72 h: 12.5–400 μg/mL). After the treatment with fucoidan, 10 µL of 5 mg/mL 3-(4,5-dimethylthiazol-2-yl)-2,5-diphenyltetrazolium bromide (MTT) (Affymetrix, Santa Clara, MA, USA) was added, and cells were incubated for 2 h at 37 °C in the dark. Next, the medium was discarded, and formazan crystals were dissolved in 100 µL DMSO (VWR, Darmstadt, Germany). The absorbance was measured at 570 nm using DeNovix DS-11 (DeNovix, Wilmington, DE, USA).

### 4.4. Flow Cytometry

#### 4.4.1. Cell Cycle Analysis

To assess the effect of fucoidan on cell cycle distribution, cells were seeded into 6-well plates. The next day, cells were treated with concentrations representing 0.5 (0.5FV), 1 (1FV), and 2-fold IC50 (2FV) doses of FV for 24 h. After that, cells were harvested, washed in PBS, fixed with cold 70% ethanol, and incubated at −20 °C overnight. Samples were stained with a PBS solution containing 20 µL 500 µg/mL RNase (Cayman Chemical, Ann Arbor, MI, USA) and 40 µL of 250 µg/mL propidium iodide (PI) (Cayman Chemical, MI, USA) in a total volume of 200 µL at 37 °C for 30 min. Data were acquired using BD Accuri C6 Plus (BD Biosciences, San Jose, CA, USA), and results were analyzed with FlowJo software (FlowJo, LLC, Ashland, OR, USA).

#### 4.4.2. Apoptosis Analysis

In order to assess the induction of apoptosis indicated by phosphatidylserine externalization following treatment with fucoidan, cisplatin or their combination, cells were stained with Annexin V Apoptosis Detection Kit I (BD Biosciences, San Jose, CA, USA) according to the manufacturer’s protocol. Briefly, cells were harvested, washed, resuspended in 100 µL of Annexin Binding Buffer, and incubated with 5 µL of Annexin V APC-conjugated antibody and 5 µL of PI for 15 min in the dark at room temperature. Next, cells were centrifuged and resuspended in 200 µL PBS. Data were acquired by means of BD Accuri C6 Plus (BD Biosciences, San Jose, CA, USA), and results were analyzed with FlowJo software (FlowJo, LLC, Ashland, OR, USA).

#### 4.4.3. ROS Production Analysis

In order to assess the level of intracellular reactive oxygen species production, the cells were treated with 0.5FV, 1FV and 2FV for 24 h, and collected and washed twice in PBS. Pellets were resuspended in 1 mL of PBS and incubated with 1 µL of 2,7-dichlorofluorescein diacetate (DCFH-DA) (Sigma Aldrich, MO, USA) at 37 °C for 45 min in the dark. Next, cells were centrifuged and resuspended in 200 µL PBS. DCFH-DA fluorescence was measured by BD Accuri C6 Plus (BD Biosciences, San Jose, CA, USA) and data were analyzed with FlowJo software (FlowJo, LLC, Ashland, OR, USA).

### 4.5. Western Blot

Total protein was extracted with RIPA lysing buffer (Sigma Aldrich, St. Louis, MO, USA). The concentration of samples was assessed by means of Pierce™ BCA Protein Assay Kit (Thermo Scientific, Waltham, MA, USA) according to the manufacturer’s protocol. Ten micrograms of denatured protein samples were separated during SDS-PAGE gel electrophoresis and were transferred onto PVDF membrane using Trans-Blot^®^ Turbo™Transfer Pack (both provided by Bio-Rad Laboratories Ltd., Hercules, CA, USA). Non-specific binding sites were blocked by 1 h incubation in 5% non-fat dry milk, and the membrane was incubated with the respective primary antibody solution in TBST: anti-CASP3 (full form) (1:1000; no. ab49822, Abcam, Cambridge, UK), anti-PARP (full form) (1:1000; no. 9542S, Cell Signaling Technology, Danvers, MA, USA), anti-BECN1 (1:750; no. ab118148, Abcam, UK) and anti- β-Actin (1:500; sc130656, Santa Cruz Biotechnology, Dallas, TX, USA) overnight at 4 °C. Next, membranes were washed three times in TBST and incubated with specific HRP-conjugated secondary antibody for 1 h at room temperature. Protein bands were visualized by chemiluminescence induced by WesternBright™ Quantum kit (Advansta, San Jose, CA, USA) using ChemiDoc Touch Imaging System (Bio-Rad Laboratories Ltd., CA, USA). The results were semi-quantified by means of ImageJ software (National Institutes of Health, Bethesda, MD, USA), and the relative protein level was normalized to mean signal intensity of reference protein β-Actin.

### 4.6. Analysis of Drug Combinatory Effect

The modified probability sum test (according to Schrader et al. [50] and Stoehr et al. [51]) was applied to assess the mode of combinatory effect, according to the formula:

q = P[A + B]/(P[A] + P[B] − P[A] × P[B])(1)

where P[A] represents the efficacy of drug A and P[B] the efficacy of drug B, while P[A + B] depicts the efficacy of a combination of both drugs at the same doses. Assessed value/expected value (sum of probability of independent events) ratios were defined: q < 0.85 as antagonism, while q = 1 ± 0.15 indicates additivity and q > 1.15 synergism.

### 4.7. Statistical Analysis

Statistical analysis was conducted using Two-way ANOVA test in GraphPad Prism Software (GraphPad Software, San Diego, CA, USA). P values lower than 0.05 were considered significant and are labeled by asterisks (*) for *p* < 0.05, (**) for *p* < 0.01, (***) for *p* < 0.001, and (****) for *p* ≤ 0.0001. All experiments have been carried out in biological triplicates.

## 5. Conclusions

In conclusion, for the first time, we proved that crude *Fucus vesiculosus* fucoidan exerts anticancer effects towards HNSCC in vitro, which are manifested by the inhibition of proliferation, cell cycle arrest, induction of apoptosis, and regulation of reactive oxygen species synthesis. Also, we observed synergy between fucoidan and cisplatin in the tested head and neck cancer cell lines. Therefore, fucoidan, as a non-toxic, well-tolerated compound poses a good candidate for a complementary agent in chemotherapy. However, additional research is crucial in order to investigate the mechanistic background behind its activity.

## Figures and Tables

**Table 1:** Analysis of drug combinatory effect.

Test	Cell Line	*q*-Value	Combinatory Effect
MTT	H103	2.90	Synergism
	FaDu	1.51	Synergism
	KB	1.24	Synergism
Apoptosis analysis	H103	1.19	Synergism
	FaDu	0.94	Additivity
	KB	1.22	Synergism

**Table 2:** The number of cells seeded for MTT assay after 24, 48 and 72 h incubation with fucoidan.

	Cell Number		
Incubation Time	H103	FaDu	KB
24 h	2000	2500	3000
48 h	1000	2000	1500
72 h	750	1000	750
